# Effect of Long‐Term Non‐Weight Bearing on the Morphology and Function of the Heel Fat Pad

**DOI:** 10.1002/jfa2.70157

**Published:** 2026-04-29

**Authors:** Toshihiro Maemichi, Masatomo Matsumoto, Toshiharu Tsutsui, Tsukasa Kumai

**Affiliations:** ^1^ Faculty of Sport Sciences Waseda University Shinjuku Japan; ^2^ Institute of Life Innovation Studies Toyo University Bunkyō Japan; ^3^ Graduate School of Sport Sciences Waseda University Shinjuku Japan; ^4^ Department of Rehabilitation Kuwana City Medicine Center Mie Japan

**Keywords:** aging, heel fat pad, macrochamber layer, microchamber layer, non‐weight‐bearing, ultrasound

## Abstract

**Background:**

We aimed to investigate the effects of long‐term non‐weight‐bearing conditions, including prolonged bedridden states, on the morphology and function of the heel fat pad.

**Methods:**

We recruited 13 elderly individuals bedridden for over 1 year (BR group) and 89 age‐matched healthy controls (CON group) living independently. Using diagnostic ultrasound imaging, we measured the thickness of the microchamber, macrochamber, and whole layers of the heel fat pad under non‐compressed and compressed conditions.

**Results:**

Significant reductions in both non‐compressed and compressed thicknesses were observed across all layers in the BR group. Furthermore, a significant negative correlation was observed between age and thickness change in the BR group, whereas no such correlation was observed in the CON group.

**Conclusion:**

Long‐term non‐weight‐bearing was associated with morphological changes and functional deterioration in the heel fat pad. These findings suggest that prolonged unloading may influence heel fat pad morphology, which may be relevant when considering weight‐bearing management.

## 1 Introduction

The heel fat pad forms a cup‐like structure that envelops the plantar, medial, lateral, and posterior aspects of the calcaneus. Within this structure, honeycomb‐like fibrous septa are filled with adipose tissue. Its primary function is to absorb shocks generated during walking and running, thereby protecting the underlying bones and joints from mechanical loads [1, 2, 3, 4]. These tissues maintain their healthy state through regular weight‐bearing stimulation, which is essential for their appropriate function. However, long‐term non‐weight‐bearing conditions, such as prolonged bedridden states or movement restrictions, may induce morphological and functional alterations in the heel fat pad; however, detailed investigations on this topic remain limited.

Muscle atrophy and decreased bone mineral density are well‐documented consequences in bedridden patients and wheelchair users [5]. Particularly, the calcaneus exhibits significant bone loss during prolonged non‐weight‐bearing, which is associated with increased risks of fractures and pain upon reloading [6]. Similarly, dysfunction of the heel fat pad has been implicated in heel pain, especially in older adults and patients with heel disorders, where degeneration and destruction of elastic fibers, reduction of healthy adipose tissue, and leakage of lipid droplets from the fibrous septa lead to thinning of the fat pad [7, 8]. Given that calcaneus bone density is maintained by mechanical loading, it is plausible that the heel fat pad also relies on mechanical stimuli to preserve its structure and function. Consequently, both tissues may undergo degenerative changes under unloading conditions, warranting further research.

The heel fat pad is anatomically divided into two distinct layers by fibrous septa: the superficial microchambers (MICs) and the deeper macrochambers (MACs). Due to differences in tissue morphology, the MAC layer primarily absorbs shocks, while the MIC layer regulates excessive deformation of the MAC layer [9, 10, 11]. Our previous studies have demonstrated that the morphology and function of MICs and MACs vary depending on factors such as age, height, weight, and the presence of heel pain. However, it remains unclear which layer is most affected by long‐term non‐weight‐bearing.

In this study, we aim to investigate in detail the effects of prolonged unloading on the morphology and function of the MIC and MAC layers of the heel fat pad. By comparing morphological changes during unloaded and loaded conditions, we sought to provide fundamental insights relevant to rehabilitation and return‐to‐sport strategies. Additionally, the findings may offer valuable information for bedridden elderly patients requiring disuse prevention and gait reacquisition, as well as athletes subjected to unloading during rehabilitation.

## 2 Materials and Methods

### 2.1 Participants

This study included 89 healthy men aged 70–80 years (169 legs, CONTROL group) and 13 men of the same age range who had been bedridden for over 1 year (26 legs, BEDRIDDEN group). Exclusion criteria were as follows: (1) engagement in vigorous physical activity or alcohol consumption within 48 h prior to measurement; (2) history of foot or ankle surgery or major trauma; (3) presence of orthopedic foot or ankle injuries including heel pain or ligament damage; (4) rheumatic diseases such as osteoarthritis, gout, or rheumatoid arthritis; and (5) systemic diseases, including diabetes or connective tissue disorders. Causes of bedridden status included stroke, gait difficulty due to aging, fractures or falls, and joint diseases. Basic demographic data, including age, height, weight, and body mass index (BMI), were collected (Table 1).

**TABLE 1:** Physical characteristics of participants.

	Age (years)	Height (cm)	Weight (kg)	BMI
CON (*n* = 89)	75.1 ± 3.9	157.5 ± 8.1	57.9 ± 8.8	23.3 ± 2.7
BR (*n* = 13)	78.6 ± 5.3	152.4 ± 11.0	47.1* ± 12.2	20.1 ± 4.3

*Note:* Data are shown as mean ± SD. * Indicates a statistically significant difference compared with the CON group (*p* < 0.05).

All participants were informed about the study objectives, measurement procedures, and ethical considerations, and provided written informed consent. The study protocol was approved by the Institutional Ethics Committee (Approval No. 2025‐520).

Measurements were obtained from both feet of each participant. Although both feet were included in the analysis, this approach has been adopted in previous studies investigating heel fat pad characteristics. Therefore, this study followed a similar methodology. Nevertheless, the potential dependency between the left and right feet was considered when interpreting the results.

### 2.2 Thickness Measurement of the Heel Fat Pad

**FIGURE 1:**
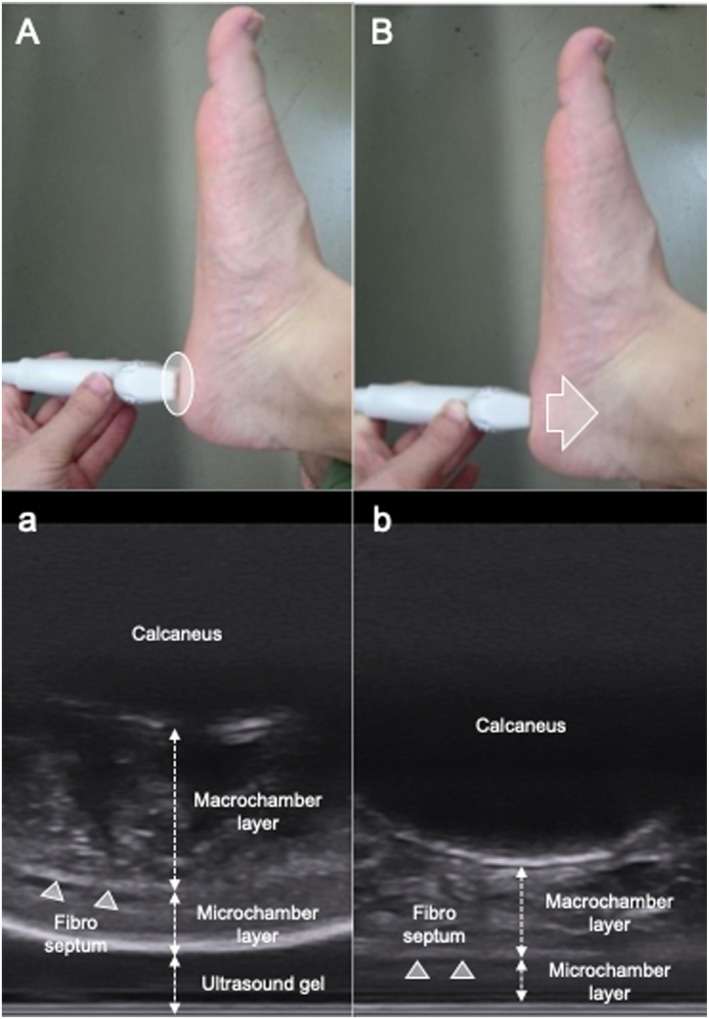
Method for assessing heel fat pad thickness using an ultrasound. (A) This is an image taken when measuring thickness without compression. Ultrasound gel was placed between the heel fat pad and the ultrasound probe to prevent the ultrasound probe from directly touching the skin and applying pressure. (a) Is an actual ultrasound image of the heel fat pad without compression. (B) This is an image taken when measuring thickness with compression. Maximum pressure was applied. (b) Is an ultrasound image of the heel fat pad with actual compression.

The thickness of the heel fat pad was measured using an ultrasound imaging device (Noblus, HITACHI, Japan) equipped with a 10 MHz high‐frequency linear probe. Following previous protocols, measurements were performed at three sites: from the inferior border of the calcaneal tuberosity to the bottom of the fibrous septum (macrochamber layer, MAC layer), from the bottom of the fibrous septum to the skin surface (microchamber layer, MIC layer), and the combined thickness of both layers (Whole layer). The MAC layer included the plantar fascia, MACs, and fibrous septum, while the MIC layer included MICs and skin (Figure 1).

Measurements were conducted under two conditions: an uncompressed state, where the probe did not contact the heel fat pad, separated by ultrasound gel (pseudo‐unloaded condition), and a compressed state, where the examiner applied maximum pressure with the probe onto the heel fat pad (pseudo‐loaded condition) (Figure 2). Three images were obtained per condition, and the mean thickness was calculated. To standardize the compression procedure, the examiner applied gradual pressure until maximal deformation of the heel fat pad was visually confirmed using ultrasound imaging. The thickness change upon compression was calculated as the difference between the uncompressed and compressed thicknesses. All measurements were performed by the same examiner, and intra‐examiner reliability was assessed by the intraclass correlation coefficient (one‐way random effects model 1, single measurement, agreement) exceeding 0.95 for all layers and conditions.

FIGURE 2(A) Relationship between uncompressed heel fat pad thickness and age, height, and weight. (B) Relationship between compressed heel fat pad thickness and age, height, and weight. (C) Relationship between the rate of change in heel fat pad thickness and age, height, and weight.

**Figure:**
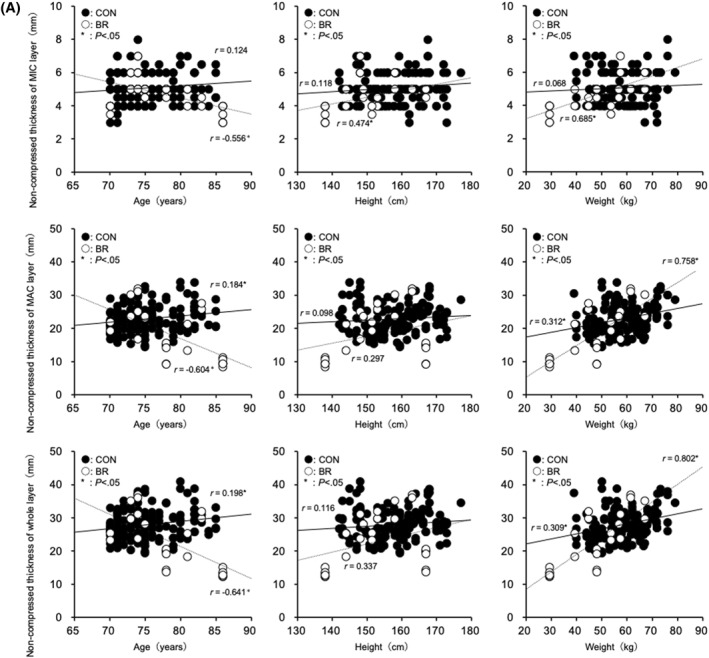

**Figure:**
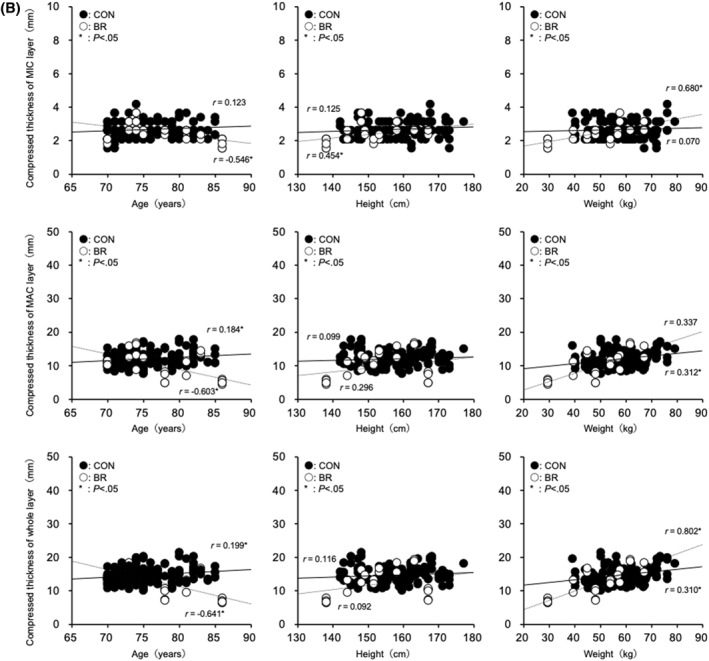

**Figure:**
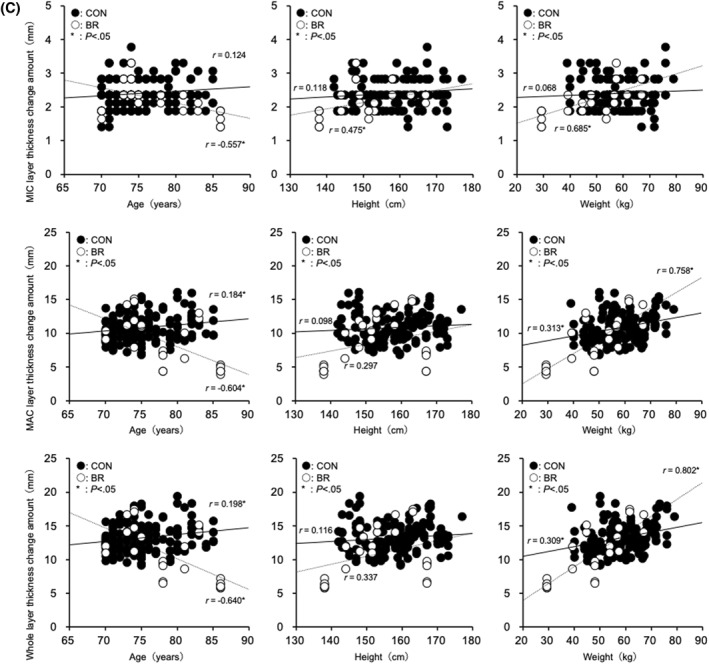

### 2.3 Statistical Analysis

Statistical analyses were conducted using IBM SPSS Statistics for Windows, version 28 (IBM Corp., Armonk, N.Y., USA). Data normality was confirmed using the Shapiro–Wilk test. Independent *t*‐tests were used to compare the uncompressed thickness, compressed thickness, and thickness change of MAC, MIC, and Whole layers between groups. Pearson's correlation coefficient was calculated to assess the relationships between thickness parameters and age, height, and weight. The significance level was set at *p* < 0.05. The substantial imbalance in sample size between the BR and CON groups is a recognized limitation; therefore, the results should be interpreted as exploratory. Additionally, although both feet were treated as independent observations, the possibility of within‐subject correlation was acknowledged.

## 3 Results

The basic physical characteristics of the participants are shown in Table 1. Regarding body weight, a significant difference was observed between the BEDRIDDEN (BR) group (47.1 ± 12.2 kg) and the CONTROL (CON) group (57.9 ± 8.28 kg; *p* = 0.001).

Significant differences were observed between the BR and CON groups in all layers of the heel fat pad (microchamber layer [MIC], macrochamber layer [MAC], and whole layer [Whole layer]) for non‐compressed thickness, compressed thickness, and thickness change (Table 2).

**TABLE 2:** Evaluation of heel fat pad thickness using an ultrasound.

		BR	CON	95% CI	*p*	Effect size (*r*)
Non‐compression thickness (mm)	MIC layer	4.6 ± 0.9	5.1 ± 0.9	0.1–0.9	0.01	0.41
	MAC layer	18.1 ± 7.7	22.8 ± 4.1	3.2–7.1	0.002	0.56
	Whole layer	22.7 ± 8.0	27.9 ± 4.3	3.6–7.7	0.001	0.57
Compression thickness (mm)	MIC layer	2.4 ± 0.5	2.7 ± 0.5	0.0–0.5	0.02	0.41
	MAC layer	9.5 ± 4.0	12.0 ± 2.1	1.1–4.4	0.002	0.56
	Whole layer	12.0 ± 4.2	14.7 ± 2.3	1.3–4.7	0.001	0.57
Amount of change (mm)	MIC layer	2.2 ± 0.4	5.1 ± 0.4	0.1–0.4	0.02	0.41
	MAC layer	8.6 ± 3.6	10.8 ± 1.9	1.5–3.4	0.002	0.56
	Whole layer	10.8 ± 3.8	13.2 ± 2.0	1.7–3.7	0.001	0.57

*Note:* Data are shown as mean ± SD.

In the CONTROL group, age was positively correlated with MAC for non‐compressed (*r* = 0.184, *p* = 0.017), compressed (*r* = 0.184, *p* = 0.017), and thickness change (*r* = 0.184, *p* = 0.017). Positive correlations were also observed in the whole layer for non‐compressed (*r* = 0.198, *p* = 0.01), compressed (*r* = 0.199, *p* = 0.009), and thickness change (*r* = 0.198, *p* = 0.01). No significant correlations were observed between age and MIC‐related variables. Body weight was positively correlated with MAC for non‐compressed (*r* = 0.312, *p* < 0.01), compressed (*r* = 0.312, *p* < 0.01), and thickness change (*r* = 0.313, *p* < 0.01), and with the whole layer for non‐compressed (*r* = 0.309, *p* < 0.001), compressed (*r* = 0.310, *p* < 0.01), and thickness change (*r* = 0.309, *p* < 0.01). No correlations were observed between weight and MIC‐related variables. Height was not significantly correlated with any parameter.

In the BEDRIDDEN group, age was negatively correlated with MIC for non‐compressed (*r* = −0.556, *p* = 0.003), compressed (*r* = −0.546, *p* = 0.004), and thickness change (*r* = −0.557, *p* = 0.003); with MAC for non‐compressed (*r* = −0.604, *p* < 0.001), compressed (*r* = −0.603, *p* = 0.001), and thickness change (*r* = −0.604, *p* = 0.001); and with the whole layer for non‐compressed (*r* = −0.641, *p* < 0.001), compressed (*r* = −0.641, *p* < 0.001), and thickness change (*r* = −0.640, *p* < 0.001). Body weight was positively correlated with MIC for non‐compressed (*r* = 0.685, *p* < 0.001), compressed (*r* = 0.680, *p* < 0.001), and thickness change (*r* = 0.685, *p* < 0.001); with MAC for non‐compressed (*r* = 0.758, *p* < 0.001), compressed (*r* = 0.757, *p* < 0.001), and thickness change (*r* = 0.758, *p* < 0.001); and with the whole layer for non‐compressed (*r* = 0.802, *p* < 0.001), compressed (*r* = 0.802, *p* < 0.001), and thickness change (*r* = 0.802, *p* < 0.001). Height was positively correlated with MIC for non‐compressed (*r* = 0.474, *p* = 0.015), compressed (*r* = 0.454, *p* = 0.02), and thickness change (*r* = 0.475, *p* = 0.014); however, no significant correlations were observed with MAC or the whole layer (Figure 2).

## 4 Discussion

The purpose of this study was to examine the effect of long‐term non‐weight bearing on the morphology and function of the heel fat pad, and healthy subjects and elderly people who had been bedridden for more than 1 year were examined.

In the BR group, significant decreases in both the uncompressed and compressed thickness were observed in the MIC, MAC, and Whole layers, and age showed a negative correlation with thickness changes (MIC layer: *r* = −0.546 to −0.557; MAC layer: *r* = −0.603 to −0.604; Whole layer: *r* = −0.640 to −0.641; *p* < 0.01). In contrast, in the CON group, age was positively correlated with the MAC and Whole layers (MAC layer: *r* = 0.184; Whole layer: *r* = 0.198–0.199; *p* < 0.05), while no significant correlation was observed in the MIC layer. Regarding body weight, positive correlations were found in the BR group across the MIC, MAC, and Whole layers (MIC layer: *r* = 0.680–0.685; MAC layer: *r* = 0.757–0.758; Whole layer: *r* = 0.802; *p* < 0.001), whereas in the CON group, positive correlations were observed in the MAC and Whole layers (MAC layer: *r* = 0.312–0.313; Whole layer: *r* = 0.309–0.310; *p* < 0.01), but no correlation was found in the MIC layer. For height, positive correlations were observed in the BR group only in the MIC layer (*r* = 0.454–0.475; *p* < 0.05), while no significant correlations were found in the MAC or Whole layers. In the CON group, no significant correlations with height were observed in any layer. These results suggest that long‐term unloading has a more pronounced effect on the morphology and function of the heel fat pad than age‐related changes. The observed positive correlations with body weight and height indicate that body composition may influence the thickness of the heel fat pad. Indeed, our previous studies reported age‐related thinning of the heel fat pad and reductions in its elasticity [8, 12]. These reports were based on healthy individuals and provided general physiological insights. Notably, in the MIC layer of the BR group, the simultaneous presence of a negative correlation with age and positive correlations with body weight and height suggests that age and body composition may have a combined effect on morphological changes under conditions of prolonged unloading.

In terms of body composition, only body weight showed a significant difference between the groups. The BR group had been bedridden for over a year, and compared to their lives when they were able to move around on their own, their physical activity and mobility levels had declined significantly compared to their prior independent status. Changes in physiological functions caused by being bedridden can be observed in bedrest experiments conducted in the fields of clinical medicine and space medicine. Greenleaf and Kozlowski observed that bed rest induces changes in body fluid distribution and reductions in plasma volume, and alterations in both electrolyte balance and nitrogen excretion [13]. Taylor et al. also conducted a three‐ to four‐week bedrest experiment on young males and reported that basal metabolic rate decreased by 8.8% [14]. It is also possible that differences in muscle mass due to the presence or absence of exercise may be affecting these changes. While those who exercise tend to maintain muscle mass, those who are in a long‐term non‐weight‐bearing state experience sarcopenia (a decrease in muscle mass due to aging) and disuse atrophy, leading to a decrease in muscle mass. This decrease in muscle mass may have led to weight loss, especially in those who are bedridden, which may lead to a decrease in muscle mass and overall weight loss.

Regarding the morphological changes in the calcaneal fat pad, there was a significant decrease in both the MIC and MAC layers. This may be because long‐term non‐weight bearing caused dysfunction of the fibrous septa in the calcaneal fat pad, leading to a decrease in adipocytes and a decrease in the elasticity of the fibers. In addition to reduced mechanical loading, physiological mechanisms may also contribute to the observed changes. Prolonged unloading has been associated with reduced peripheral blood flow and metabolic alterations in adipose tissue. These changes may impair adipocyte function and promote degeneration of fibrous septa within the heel fat pad. Such mechanisms could explain the structural thinning observed in the present study. Previous studies have reported that in elderly people, elastic fibers in the calcaneal fat pad deteriorate and tissue thinning progresses, which is said to cause a decrease in the ability to adapt to mechanical loads [8, 12, 15]. Furthermore, a negative correlation was observed between age and thickness change in the BR group, suggesting that morphological changes due to non‐weight bearing are even more pronounced in elderly people. This change is thought to be due to the fact that the metabolic ability of the heel fat pad decreases with age, making it difficult to repair and maintain. The decrease in thickness of the MAC layer indicates a decrease in the heel fat pad's inherent shock‐absorbing function. The MAC layer, which is the deep layer of the heel fat pad, mainly absorbs shock and reduces damage to bones and joints, but it is thought that its function decreases with the deterioration of fat tissue when the fat pad is left unloaded. This decrease in function may increase the risk of heel pain and fatigue fractures when weight is applied again. This risk is particularly significant in elderly people and bedridden patients, and careful load management is necessary during rehabilitation. Similarly, it was confirmed that the thickness of the MIC layer decreased when the fat pad was unloaded. The MIC layer is located in the superficial layer of the heel fat pad and mainly plays a role in controlling excessive deformation of the deep layer, but its elasticity decreases in the unloaded state, and the loss of fat tissue may progress. This change may cause the heel fat pad to not function properly when walking or running, causing pain and fatigue. This study demonstrated that the effect of non‐weight bearing on the heel fat pad provides important insights for clinical practice. It became clear that heel weight‐bearing management is essential, especially for elderly people, long‐term hospitalized patients, and athletes undergoing rehabilitation. In the future, it will be necessary to develop intervention methods and rehabilitation programs to suppress changes caused by non‐weight bearing.

## 5 Limitations

Because the sample size of the bedridden group was relatively small, caution is required when generalizing the results. The recruitment of bedridden elderly individuals who met the inclusion criteria was challenging, which contributed to the imbalance in sample size between the groups. In addition, detailed data on the subjects' activity levels and living environments were insufficient, and a detailed examination of their effects on the heel fat pad was not possible. Furthermore, this study only evaluated the effects of non‐weight bearing on the heel fat pad and did not include data on the long‐term effects or recovery process. Therefore, larger studies and long‐term observations are needed in the future.

## 6 Conclusion

This study observed the effects of long‐term non‐weight bearing on the morphology and function of the heel fat pad. It was confirmed that the thickness of the MIC and MAC layers was significantly reduced in the BR group, and that elasticity was also reduced. These findings suggest that prolonged unloading may influence heel fat pad morphology and may be relevant when considering weight‐bearing management strategies.

## Author Contributions

**Toshihiro Maemichi:** conceptualization, formal analysis, investigation, methodology, project administration, writing – original draft. **Masatomo Matsumoto:** conceptualization, methodology, writing – review and editing. **Toshiharu Tsutsui:** conceptualization, formal analysis, investigation, methodology, writing – review and editing. **Tsukasa Kumai:** supervision, writing – review and editing.

## Funding

The authors have nothing to report.

## Disclosure

The authors have nothing to report.

## Conflicts of Interest

The authors declare no conflicts of interest.

## Data Availability

The data that support the findings of this study are available from the corresponding author upon reasonable request.
